# “We have to save him”: a qualitative study on care transition decisions in Ontario’s long-term care settings during the COVID-19 pandemic

**DOI:** 10.1186/s12877-023-04295-1

**Published:** 2023-09-26

**Authors:** Sarah Carbone, Whitney Berta, Susan Law, Kerry Kuluski

**Affiliations:** 1 Institute of Health Policy, Management and Evaluation, University of Toronto, 155 College St, Toronto, ON M5T 3M6 Canada; 2 Institute for Better Health Trillium Health Partners, 100 Queensway West, Mississauga, ON L5B 1B8 Canada

**Keywords:** Aging, Long-Term Care, Care Transition, Community Care, Decision-Making, COVID-19, CRISIS

## Abstract

**Background:**

The COVID-19 pandemic has contributed to a global crisis in long-term care (LTC) with devastating consequences for residents, families and health professionals. In Ontario, Canada the severity of this crisis has prompted some care partners to move residents home with them for the duration or a portion of the pandemic. This type of care transition, from LTC to home care, was highly unusual pre-pandemic and arguably suboptimal for adults with complex needs. This paper presents the findings of a qualitative study to better understand how residents, care partners, and health professionals made care transition decisions in Ontario’s LTC settings during the pandemic.

**Methods:**

Semi-structured interviews were conducted with 32 residents, care partners and health professionals who considered, supported or pursued a care transition in a LTC setting in Ontario during the pandemic. Crisis Decision Theory was used to structure the analysis.

**Results:**

The results highlighted significant individual and group differences in how participants assessed the severity of the crisis and evaluated response options. Key factors that had an impact on decision trajectories included the individuals’ emotional responses to the pandemic, personal identities and available resources.

**Conclusions:**

The findings from this study offer novel important insights regarding how individuals and groups perceive and respond to crisis events.

## Introduction

The COVID-19 pandemic has been a global crisis with impacts on every level of society [1]. In many countries, these impacts have been disproportionate to long-term care (LTC) sectors, which have long struggled with infrastructure and workforce challenges [2] and where the complex health status of residents have made them particularly vulnerable to severe COVID-19 infection [3]. In Ontario, Canada hundreds of outbreaks have been recorded in LTC and more than 60% of all COVID-19 deaths in the province of Ontario have been among LTC residents [4]. Although the distribution of vaccines reduced both infections and deaths [4], LTC homes have continued to grapple with frequent outbreaks, exacerbating existing staffing challenges [5] and forcing many residents to be confined to their rooms for prolonged periods of time [6, 7]. Various public health measures have been implemented in Ontario to protect the LTC population, including restricting staff employment across homes and preventing non-essential visitors from entering the homes [8]. Unfortunately, these measures have negatively impacted the quality of life of many LTC residents and their care partners and created new risks including increased isolation, deterioration, and stress [6, 7].

There is evidence that the COVID-19 pandemic has increased public awareness of the risks in LTC and led Ontarians to reconsider aging in these settings [9]. Not only have Ontarians shown less interest in entering LTC in the future [9], some care partners decided to move residents home with them for the duration or a portion of the pandemic [10–12]. This type of care transition, from LTC to home care, was highly uncommon prior to the pandemic occurring in less than 1% of Canadian LTC residents [13, 14]. This is in part due to the significant risks of transitioning an older adult, whose high care needs make them particularly vulnerable to adverse health events or outcomes [15, 16]. In addition, care transitions from LTC to the community may have been undesirable or unfeasible due to high levels of caregiver burnout and limited access to home care resources [17]. Finally, the transitions require complex coordination between multiple stakeholders, including the resident, their care partner, and the health professionals from the LTC and community care sectors who would all be impacted by the decision. Yet, despite the potential consequences and complexities of this type of care transition, it appears that the COVID-19 pandemic has presented a significant enough crisis in LTC that many Ontarians are looking to age elsewhere.

Crisis Decision Theory (CDT) may offer a useful lens to understand this apparent shift in care transition decision-making during the pandemic. Initially proposed by Sweeny (2008), CDT describes the cognitive processes that people go through when they experience a crisis event [18]. Within the theory, crisis is defined broadly as any “negative event that commands a person’s attention” (18; p.61). According to Sweeny (2008), people go through a three-stage process to respond: (1) assessing the severity of the negative event; (2) determining response options; and (3) evaluating response options [18]Overall, the theory focuses on understanding both the decision processes that occur during a crisis, and the factors that predict people’s choices. Parts of the theory have been previously applied to help understand individual responses and behaviours to a variety of crises including disease outbreaks [19]. Researchers have also discussed the theory in the context of the COVID-19 pandemic, to explore crisis responses in multiple industries [20, 21]. To date no research has been done to explore CDT in the context of LTC transition decisions during the pandemic.

For years prior to the pandemic, Ontario’s LTC settings had become regarded as spaces where older adults would reside until they die [13]. Thus, we know relatively little about the reasoning behind a decision to move a resident out of LTC, even in normal circumstances. Despite the complexity and risks involved with this type of care transition, the COVID-19 pandemic appears to have motivated Ontarians’ to pursue care transitions back to the community. This paper presents the findings of a qualitative study to understand how residents, care partners and health professionals made care transition decisions in LTC settings in Ontario during the COVID-19 pandemic, analyzed through the lens of CDT.

## Methods

### Study design

Qualitative description was used to explore stakeholders’ transition decision-making processes during the COVID-19 pandemic. Rooted in naturalistic inquiry, qualitative description is a research approach often used to enhance our understanding of complex experiences or events [22]. It aims to provide straightforward, low-inference descriptions that closely resemble the original data [23]. Analyses may be completed inductively, deductively or using a blended approach [23]. For this study, we used both inductive and deductive approaches to explore stakeholders’ reports of their decision-making processes, and applied the lens of CDT in our analysis. Philosophically, qualitative description is aligned with social constructivism which recognizes the subjective and varied nature of human experiences [24, 25]. In social constructivism, researchers position themselves in the research, recognizing that their own experiences and backgrounds shape their interpretations of the data [24, 25].

### Research team

The study team comprised a doctoral candidate (SC) and several senior researchers (KK, SL, WB) with expertise in aging, LTC and patient and family engagement. All researchers were trained in qualitative research methods and had prior experience conducting health research. Data were collected by one researcher (SC) with no prior connection to the participants; however, all researchers were involved in developing the data collection materials and interpreting the data. Some members of the study team had prior experience as a care partner in a LTC setting; however, none were caring for a resident during the pandemic. The team’s interest in the topic was inspired by the personal narratives reported in the news of LTC residents and care partners impacted by the pandemic.

### Crisis decision theory

**Fig. 1:**
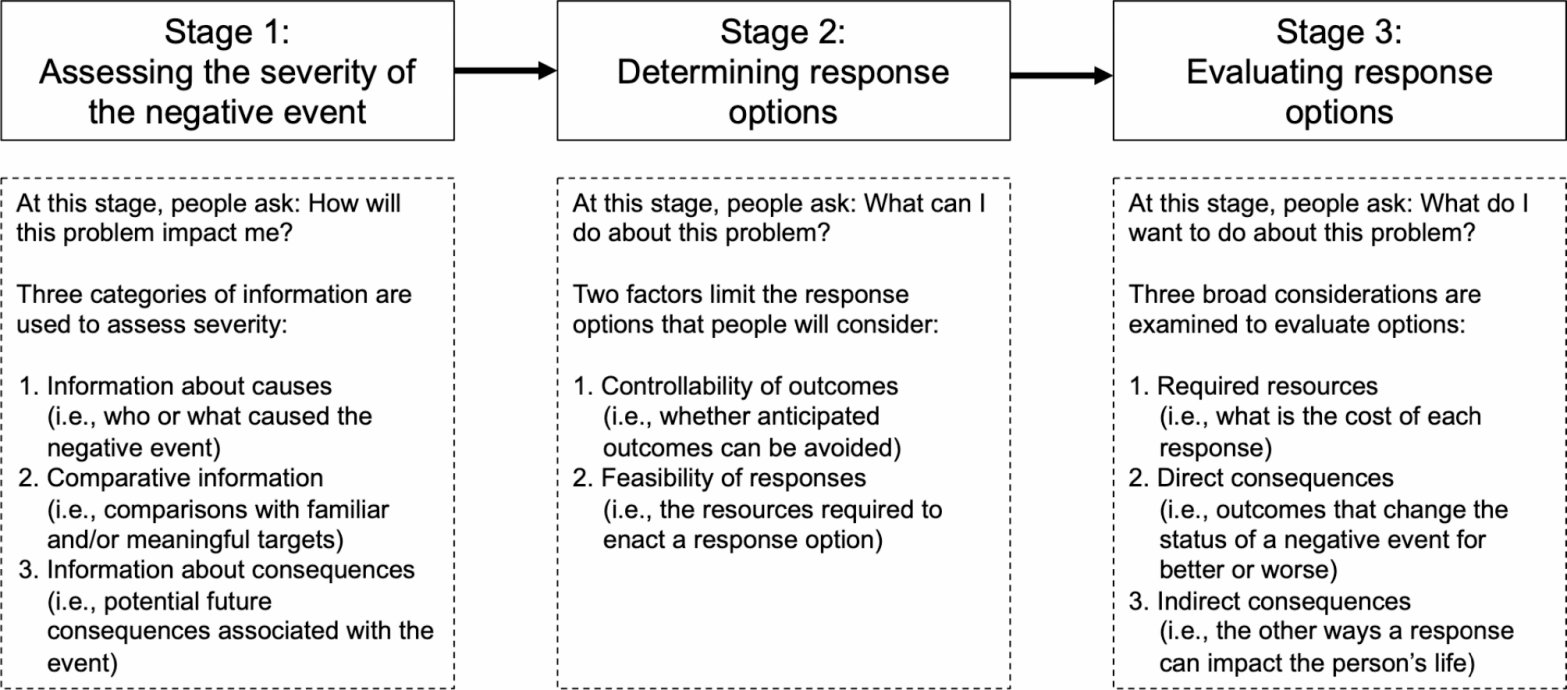
Crisis Decision Theory * Revised and reproduced from Sweeny K. Crisis decision theory: Decisions in the face of negative events. Psychological Bulletin. 2008;134 [1]:*61–76.*

CDT was used to structure and interpret this research [18] because it offers a straightforward framework through which to understand decision processes with an emphasis on stress and coping (Fig. 1). The theory links existing theories on stress and coping with decision research to describe the cognitive processes used by individuals to respond to a crisis event. According to Sweeny, people go through a three-stage process when responding to a crisis:

1. Assessing the severity of the negative event (i.e., determining how the event will affect their lives and whether there is a need to respond);
2. Determining response options (i.e., identifying what they can do about the problem); and.
3. Evaluating response options (i.e., selecting the best response based on their personal perspective and subjective criteria).

Within each stage, individuals consider a variety of information that allows them to imagine potential consequences or outcomes associated with the event, understand to what extent these outcomes are controllable, and which actions are most desirable to pursue. Individuals may go through each stage in order, may revisit earlier stages, or may become ‘stuck’ at a particular stage.

### Setting and participants

Participants were recruited from LTC settings across Ontario between December 2021 and June 2022. In Ontario, there are 627 licensed LTC homes with approximately 79,000 beds [26]. Prior to the pandemic, the sector was highly overburdened with roughly 35,000 people waiting in the community or hospital for an average of 149 days for a bed in LTC [27, 28]. However, LTC in Ontario as well as across Canada has faced significant issues for more than two decades [29], including poor physical infrastructure and inadequate staffing [30, 31]. These challenges were exacerbated during the pandemic to the point that the Canadian military was deployed to support care in some of Ontario’s LTC homes [32]. When compared with other Organization for Economic Co-operation and Development (OECD) countries Canada recorded the highest proportion of LTC deaths during the pandemic [33], and Ontario had the most outbreaks of any province or territory [34]. Thus by many standards, Ontario’s LTC sector has been a sector in crisis.

**Table 1:** Key concepts and definitions

Concept	Definition
Crisis	A negative event that commands a person’s attention.
Long-term care	An institutional setting where adults live and receive support with their health and daily living needs (for the purposes of this study, refers to both long-term care homes and retirement homes).
Community care	Services and supports provided to people at home, rather than in an institutional care setting.
Resident	A person who currently lives, or has recently lived in a long-term care setting.
Designated care partner	A person (often a family member or friend) who provides unpaid care to another person.
Health professional	A person employed in the health care system who provides medical or supportive services to a patient. For the purposes of this research, health professionals’ roles involved supporting residents and families through a care transition.

A purposeful sampling technique was used to identify participants from three stakeholder groups: residents, care partners, and health professionals (Table 1). These groups were selected due to the significant impact that transition decisions can have on their lives and work, and thus the expectation that they would be involved in transition planning. Participants were eligible if they had considered, supported, or experienced a transition out of a LTC setting in Ontario and into a home in the community during the COVID-19 pandemic. To form a comprehensive understanding of stakeholders’ transition decision-making processes we aimed to recruit a diverse range of participants from different age groups, sexes, ethnicities and geographic regions across Ontario. Recruitment was discontinued when sufficient information power was achieved [35] and the research team agreed that a comprehensive understanding of the topic had been obtained. Participants were not offered any remuneration for their participation.

Participants were recruited virtually with the support of multiple health organizations operating in Ontario, Canada. Consenting health organizations (e.g., Ontario Caregiver Organization; Ontario Long-Term Care Clinicians; Family Councils Ontario) distributed study information and recruitment flyers through their networks and on social media. A dedicated Twitter account was also created for the study to share recruitment information and organizations were encouraged to comment or share the messages from this account when appropriate. Interested participants were invited to contact the research team directly if they wished to be interviewed. Some participants were identified through snowball sampling.

### Data collection

Data were collected through interviews and a short demographic questionnaire. All interviews were conducted one-on-one, with the exception of two participants who preferred to be interviewed together. A trained researcher (SC) conducted and recorded all of the interviews by phone or videoconference. Semi-structured interview guides were used to guide the discussions and participants were encouraged to direct the course of the conversation as desired (Table 2). The interview guides focused on exploring stakeholders’ perspectives on the COVID-19 pandemic in LTC, and their care transition decision-making. The demographic questionnaire was administered at the time of the interview, and included questions on the participants’ stakeholder group, age, sex, ethnicity, and care setting. Participants were also asked to share whether they lived in an urban, suburban, or rural region of Ontario. After each interview, the researcher summarized the content of the discussion and identified key points of interest in an analytic memo [24]. Memos were reviewed periodically to identify further question prompts for the interview guide. Finally, each interview was transcribed using a secure online transcription program [36]. Identifying information was removed prior to analysis.

**Table 2:** Sample interview guide

	Interview question
1.	Tell me about yourself and your background.
2.	What was living in long-term care like before the pandemic?
3.	What types of care or support did you receive?
4.	What was living in long-term care like at the start of the COVID-19 pandemic?
5.	Would you describe the COVID-19 pandemic as a crisis?
6.	What made you consider leaving long-term care?
7.	What influenced your decision to stay in, or move from, long-term care?
8.	Was there a key moment or ‘tipping point’ when you decided to leave long-term care?
9.	Did you talk with anyone else or receive any support in making your decision?
10.	Did you receive any support or resources to help with the care transition?
11.	Would you say your decision was relatively easy or hard? Why?
12.	How is everything going for you now?
13.	Do you have any advice for others who are considering leaving long-term care during the pandemic?

### Data analysis

We analyzed the data using directed content analysis, whereby existing research or theory is used to structure coding (Hsieh, 2005). Content analysis is particularly useful when there is a considerable amount of textual data [37]. It offers a straightforward description of participant experiences to provide a comprehensive understanding of a topic from multiple perspectives [25]. CDT was used to guide analysis [18]. An initial codebook was developed with codes representing each of the three stages of CDT and their eight response predictors. Additional codes were generated by the research team to capture decision-making elements not previously identified in CDT. Overall, our aim to was understand participants’ experiences and perspectives with transition decision-making during the pandemic, and we used CDT as a theoretical lens to facilitate this understanding.

Analysis was completed in several stages. One researcher (SC) began by reviewing all the analytic memos and coding individual transcripts line by line. The initial findings were then discussed by the research team and the codebook was refined. A second round of coding was then completed alongside a senior researcher (KK) who reviewed and coded a random selection of transcripts to ensure agreement and consistency. Once coding was completed, transcripts were analyzed by code and by stakeholder group to identify nuances in individual and group perspectives. To establish trustworthiness, the authors met to discuss and refine interpretations and an audit trail was kept to document analytic decisions. NVIVO 12 software was used to store, code, and compare transcripts [38].

### Ethical considerations

Ethical approval for this study was obtained from the University of Toronto’s Health Sciences Research Ethics Board (ref: 00041349).

All participants provided written or verbal consent prior to participating in the study and ongoing assent was confirmed at various points during the interviews. The University of California, San Diego Brief Assessment of Capacity of Consent (UBACC) screening tool was used to screen decisional capacity among resident participants during the consent process [39]. One individual whose capacity was unclear was excluded from the sample.

## Results

A total of 32 individuals participated in the study, including 18 care partners, 3 residents and 11 health professionals (Table 3). Participants lived in a range of urban (n = 19) suburban (n = 5) and rural (n = 8) regions from across Ontario. Participants had made a range of care transition decisions and over half (56%) of care partners had moved a resident out of LTC during the pandemic. Health professionals had supported a range of residents and care partners, of whom some had decided to transition while others did not. From the interviews it was clear that participants’ decision-making processes were shaped by the pandemic context and their understandings of the evolving crisis circumstances in LTC. The decision-making processes that they described were also well-aligned with CDT. Below, we describe participants’ decision-making processes in depth, organized according to the three stages in CDT. Although each stage is presented separately to enhance clarity, participants often considered multiple stages simultaneously. A description of the different stages and associated response predictors with illustrative quotes is presented in Table 4.

**Table 3:** Participant characteristics (N = 32)

	Caregiver (n = 18)	Resident (n = 3)	Health Professional (n = 11)
Sex			
Male	6	2	3
Female	12	1	8
Age			
30–39	0	0	1
40–49	2	0	6
50–59	5	0	2
60–69	7	1	1
70–79	4	2	1
Ethnicity			
South Asian	1	0	1
White	17	3	8
Mixed Heritage	0	0	1
Persian	0	0	1
Region in Ontario			
Urban	11	1	7
Suburban	3	2	0
Rural	4	0	4
Institutional Setting Type			
LTC	13	3	9
Retirement Home	5	0	0
Community	0	0	2

**Table 4:** Descriptions and exemplar quotes of crisis response predictors

Stage	Response predictor	Description	Participant quotes
1. Assessing severity	Information about causes	Understandings of what caused the crisis event to occur. In some cases, individuals may feel responsible for causing the crisis themselves, or the cause may be external to them.	• “And I could see how caregivers can burn out. Because they’re just so driven by guilt, to do more, do more, do more to it, to make sure they’re happy to make sure you know, to compensate for placing them in a home.” (CP 03) • “…we understood the issue, which was COVID. Right. And no one at that time knew how really to address it” (CP 22)
	Comparative information	Existing information that individuals consider in comparison to the event as a way of approximating severity. For example, individuals may draw on their own or peers prior experiences (mental schemas). They may also draw on social comparisons to others in better or worse situations than them. Finally, they may imagine alternative outcomes.	• “…my husband did a lot of eldercare when he first graduated. And so he knew what it was all about.” (CP 03) • “I asked a lot of questions to a lot of people, and then I made my decision.” (CP 23) • “There’s some people there just the fear, they see it on TV, it’s like, I’m getting my family member out of there” (HP 30)
	Information about consequences	Understandings of the potential short- and long-term consequences of the crisis event.	• “The risks of your loved one dying in a congregate facility was very, very high. And contagion was, and we know this to be true contagion was very high rates of contagion were very high.” (CP 01) • “…We knew it was coming. We had put certain things in place, but it was like getting hit with a tsunami. I mean, it was just terrible.” (HP 02) • “I took her out. So because I knew what was coming.” (CP 12) • “But then we knew that COVID was exploding, right. And we were like, well, she could be getting it at any time.” (CP 25)
2. Determining options	Controllability of outcomes	The extent to which an individual believes that they can control the anticipated outcomes of a crisis event.	• “I know there’s like now I’m at the point where I’m thinking people, like it’s almost impossible to not get this new variant. So sooner or later, I think almost everybody’s gonna get it. So I have to take that into consideration.” (CP 08) • “What you’re telling me is BS, you know, they’re not going to… some aren’t going to come in… they are going to be very short staffed. That’s what happened to a lot of people they were left lying in their waste for days. A total mess.” (CP 23)
	Feasibility of responses	A determination of which response options are possible to execute. This often involves consideration of the resources (e.g., time, money, etc.) that would be required to pursue a response option.	• “But there are so few people available, though, to fill that role, that there’s no guarantee that you could fill a vacancy there, if even if you wanted to hire private help, and you had the wherewithal to do it. Staff, just the number of people aren’t there.” (CP 04) • “…and we considered that we and when you asked what influenced our decision is just wasn’t feasible on many levels.” (CP 05) • “I couldn’t do it… back four years ago, when he went in as a crisis admission, I was already just getting really stretched to the limit. And a lot of those community supports were there anymore.” (CP 07) • “It’s very doable, I think.” (CP 15) • “We felt we didn’t have an option.” (CP 17)
3. Evaluating options	Resources required	Consideration of the resources that the individual is willing to spend to pursue a response option.	• “But I think that it’s important to be prepared for a much longer period of care than you necessarily may envisage, and to have supports in place. The supports in place in the pandemic are probably not as great as with when you’re not in a pandemic situation” (CP 01) • “So I would have to have had home care, which wasn’t available.” (CP 03) • “It’s, it’s very expensive. I don’t think they also know how much it costs to provide long term care in their home it with what’s been paid for by the government in Ontario. They don’t, they don’t, they don’t know that actually, that they’re paying is only a fraction of what it really costs. If you’re moving into the community, you’re gonna lose a lot of services that you are already getting subsidized and paid for by the Government.” (HP 30)
	Direct consequences	Outcomes that change the status of the negative event for better or for worse.	• “And I was confident about the care he was given” (CP 03) • “And I wondered, more recently, and part of the problem is, is the policy if, if I take her out overnight now she will be discharged. That’s their policy. They were discharged. There’s no flexibility for somebody to be out for a short period of time to take her away from this unsafe environment.” (CP 04) • “I felt a safety issue in this house that I couldn’t do anything about at that point. It just there was it just didn’t make sense. It would have been unsafe.” (CP 07) • “So I do know of a couple of cases, especially when the pandemic started, where families took their loved ones home, because they were terrified. And absolutely, I understand all those cases ended up coming back to long term care, and horrible shape, because families completely underestimated how much care that person needed. Okay, I do know of one it’s because of the family taking them home for a couple months, I’m sure that hastened their death 100%.” (HP 32)
	Indirect consequences	Consideration of the other ways that a chosen response option can impact the individuals life.	• “So to take somebody like that out of their environment, and to be able to, I mean, you can’t work and take care of them that, you know, that wouldn’t work. If you’re working son or daughter. That’s it, you have to quit your job, and you’ve decided to take care of mom or dad.” (HP 02) • “So this is the thing is home care you need. Like, you can’t do it yourself. I mean, you can, but you’re not gonna have a life.” (CP 09) • “It was my family also saying it’s not reasonable, house isn’t the right place.” (CP 28)

CP = Care Partner; HP = Health Professional; R = Resident.

### Stage 1: assessing the severity of the negative event

Participants assessed the severity of the crisis by drawing on a variety of comparative information. Their affective responses played a key role in assessing severity.

The COVID-19 pandemic was recognized by participants as a constellation of crises. The first few weeks of the pandemic were characterized by a significant period of uncertainty on the disease itself and its potential impact on LTC. As one care partner described: “the whole world was running scared” (CP 17). Although information was regularly released to the public, uncertainty persisted over time: “I think it was the lack of knowledge, honestly, because we didn’t have it. And they didn’t have it. And not even in the media, the government updates we were getting…I don’t think anybody expected it to be as big as it was” (HP 02). Even months into the pandemic, participants grappled with uncertainty as new waves of COVID-19 arose and public understanding of the disease shifted. Outside of LTC, participants experienced the crisis in other areas of their lives. Care partners shared details on how the pandemic had disrupted their work lives and personal responsibilities. Meanwhile, health professionals commented on how the pandemic was a broader crisis in their communities: “So it was a crisis in the sense that, you know our patients were in crisis, our clients were in crisis, our homes were in crisis” (HP 16).

Participants described multiple anticipated consequences of the pandemic. In general, their dominant concern was of the residents contracting COVID-19. The perceived severity of this consequence often triggered the crisis decision-making process: “That life or death, that fight or flight response really came into play” (CP 12). The public health measures adopted to reduce the spread of COVID-19 in LTC settings led to additional consequences and further amplified uncertainty. For example, isolation restrictions whereby care partners were prohibited from entering LTC settings compounded stress: “…it was extremely stressful. Caregivers were calling everybody trying to figure out what our rights were. Why is this going on? Why can’t we go in? You know, what are the new rules? Why are they doing this?” (CP 05). Participants believed that these measures also inadvertently created new threats to residents’ wellbeing: “They’re either going to die of COVID, or they’re going to die of loneliness, it’s going to be one or the other” (CP 03). Other public health measures restricting health professional employment across settings exacerbated staffing challenges and raised concerns over quality of care. Together, the combined threat of the pandemic and public health responses created competing crises leading participants to ask: “where do you draw the line between keeping people safe and having a sort of quality of life” (CP 08).

To assess the severity of the crisis, participants considered a variety of comparative information. Care partners and health professionals in this study generally had multiple years of experience drew on this knowledge: “And there are instances where family are scared because something has happened in the past somewhere else in the facility or in home care” (HP 32). In some cases, they had been exposed to prior outbreaks in the LTC setting: “…every year they would have stuff like gastro outbreaks or a flu outbreak and they never could contain those before” (CP 22). Participants also made a variety of social comparisons to better understand the threat that they faced. They connected with family members, peers, support groups and health professionals to better understand the situation: “So you really have to calm down and think it through and talk it through with other people” (CP 08). In some cases, care partners’ responses were contingent on the reactions of the resident: “I think if [the resident] had been distressed and phoning and crying and begging for family, this would be a completely different situation” (CP 28). Many participants also acknowledged the media as an important source of comparative information. Participants referred to news and radio sources to gain insight towards how residents and families were grappling with the pandemic, and to read expert recommendations for protecting residents from COVID-19 infection. Health professionals expressed frustration at the overly negative portrayal of LTC in the media believing that it contributed to a more biased risk assessment: “And you know the media, in usual form, was fanning the flames and scaring everyone to death” (HP 11). Participants from all three groups appeared to agree that the negative portrayal of LTC in the media contributed to heightened concerns.

Participants’ affective responses appeared intimately related to how they assessed severity and the need to respond. Care partners expressed a range of negative emotions (e.g., fear, guilt) that guided their decision-making. Some believed that these negative emotions hindered their ability to assess risk: “So I think people do need to assess the risk and have a means of doing that, that’s sort of very realistic, and that’s based on fact and not just emotion” (CP 28). Health professionals, too, noted how care partners often struggled to differentiate between real and perceived risks of the pandemic. Some care partners had complex negative emotions related to LTC even prior to the pandemic. Several described intense feelings of guilt over their earlier decision to place a resident in LTC which amplified their sense of personality responsibility over the residents’ safety and wellbeing: “…sometimes I feel its driven by guilt and I think in this case it was like if they die because I’ve put them there that’s on [their] soul” (HP 31). Ultimately, participants’ emotions appeared to have a compounding effect on the perceived severity of the crisis, in that their initial risk assessment gave rise to negative emotions which further amplified perceived risks.

Participants exhibited diverse understandings of the pandemic; however, some trends were observed in how different groups assessed the severity of the crisis. When compared with health professionals and residents, care partners appeared to assess the crisis as most severe and felt the greatest need and urgency to respond. Many care partners described the pandemic as a life or death situation for the resident: “I did want to move her out, because of COVID, because I thought she would die” (CP 09). In contrast, residents described feeling safe in LTC: “and I remember asking her [‘do you feel safe here’] and I know that she said ‘yes’” (CP 28). Rather, residents’ concerns were typically focused on isolation, as many had spent extended periods restricted to their rooms: “…my mom sometimes would start to say ‘I’m in prison’” (CP 10). Finally, although health professionals acknowledged the concerns of residents and care partners, their risk assessments were more moderate: “…its as safe here [in LTC] as anywhere else” (HP 11). This was especially true for health professionals working in northern or rural regions where the prevalence of COVID-19 was more limited. Some health professionals’ concerns were also lessened because they had worked through prior outbreaks like the 2003 SARS pandemic. Due to these group differences some health professionals described their initial role in transition planning as one of de-escalation.

### Stage 2: determining response options

Once participants had formed an initial assessment of the severity of the crisis they began to explore opportunities to address the perceived consequences. Participants considered two potential responses: leaving the resident in LTC or moving them out of LTC, and decisions often depended on their individual circumstances.

Participants generally believed that it was possible to control the anticipated consequences of the pandemic and considered two main decision trajectories. The first option was for the resident to continue living in LTC throughout the pandemic (i.e., passive response). For residents and care partners, this option limited their perceived control because they were subject to the public health measures and precautions taken by the LTC settings. The second option was to move the resident out of LTC and into the community (i.e., active response). This option offered residents and care partners greater control because they could take the precautions that they felt were necessary. For most participants, this option involved moving the resident in to the care partners’ home. In some cases, care partners explored options to rent homes that would be more suitable to the needs of the resident: “…we looked at possibly renting a home…trying to get one level etc” (CP 05). Residents also considered moving in with their care partners; however, for one resident this was impossible, leading them to briefly consider living homeless.

The feasibility of moving a resident out of LTC during the pandemic differed across participants. Participants described many resources needed to safely transition, including: time, money, professional support, and an accessible home. Some care partners were confident that they had the ability and resources in place to support a successful transition: “my husband and I decided that we did have the space and we did have the ability to have [resident] here” (CP 06). For others, resource constraints limited the feasibility of moving the resident: “I kept mapping it out in my mind, what would I need? How would I do it? And I could solve every problem except a doctor” (CP 08). In some cases, these constraints were insurmountable: “I desperately tried to get [resident] out of there…there’s just so many things you need to take care of that we couldn’t do it” (CP 09). Health professionals believed that residents and care partners often underestimated the resources needed to transition. Despite this, they worked to advise and support the residents and care partners in accessing the resources needed to transition safely: “so we entertain all options to make sure that wherever they go, if its another home or its to the community, then it’s a safe transition” (HP 16).

### Stage 3: evaluating response options

At this stage, participants weighed the benefits and consequences of possible response options. The focus of their decision-making was broadened to consider the indirect consequences of each response.

Participants thought carefully about the different care trajectories prior to deciding. This involved evaluating the anticipated benefits and costs of each option and forming a contingency plan. Participants recognized the importance of being ‘realistic’ and not basing decisions around their emotional responses. As one care partner described: “the biggest thing is don’t have a romantic view about it. Don’t think you’re going to bring them home and all the problems are going to disappear, think long-term” (CP 03). The focus on practicality was particularly prominent in health professionals’ interviews as they tried to ensure that residents and care partners fully considered the consequences of a transition. In general, participants understood that the decision was complex, and there was no ‘right’ answer: “But I think people need to weigh the options, and really understand the consequences of what each of those decisions will be…there’s no easy answer and the answer will be different for different people” (CP 04). For some, uncertainty over the ‘right’ option led them to continue with the status quo: “Not knowing if the grass would be greener was one of the things that kept me” (R 13).

There was often a trigger event that forced participants to respond to the crisis. This trigger event was nearly always an outbreak in the LTC setting. Participants who experienced this event early in the pandemic, when uncertainty was at its highest, described having limited time to make and evaluate their decision. For example, one care partner recalled theirs and their spouses’ reaction upon learning of an outbreak: “…we looked at each other and said ‘ok we have to save him’” (CP 17 & CP 18). Other participants whose LTC settings were unaffected by the pandemic for several months had more time to formulate a plan: “I guess as a whole family…we determined that if that would happen, where there was an outbreak, we kind of had a game plan ready…she would get out of there” (CP 22). Thus, some participants had a truncated evaluation of response options due to the timing of the crisis unfolding.

A resident staying in LTC was generally associated with negative consequences. Most care partners believed that all residents living in LTC would eventually be exposed to COVID-19. In contrast, health professionals tried to reassure residents and care partners that they could control the spread of the virus. However, according to one care partner this belief shifted over time: “at the time, they felt that they could contain the outbreak…they changed their tune after. We brought her back after the outbreak was over and they said it was horrific” (CP 22). Participants also believed that residents who stayed in LTC would be forced to isolate: “the residents were isolated in their rooms, and that’s what I was worried was going to happen” (CP 12). In addition to lowering the residents’ quality of life, care partners were concerned that prolonged isolation would lead to an overall deterioration in their health. Staffing in the homes created additional concerns around quality of care. Participants from all three groups commented on how existing staffing challenges had been exacerbated during the pandemic: “they’re at the mercy of the staff, or lack of staff, they’re at the mercy of the situation” (CP 03). Despite this, some participants were confident in the care provided in LTC, even during the pandemic. As one resident stated: “well basically, this probably is the best place for me…the medical attention is far superior” (R 27).

Deciding to move a resident out of LTC was often viewed more favourably by residents and care partners; however, the option brought about its own set of challenges. Care partners generally believed that they could reduce the threat of COVID-19 by moving the residents; however, knew that risk could not be eliminated entirely due to community spread: “For the most part, I guess I can’t protect her totally right. I mean, but I figured I could protect her more than her being in there” (CP 23). Residents and care partners also believed that the residents’ quality of life would improve following a care transition. By caring for the resident in their homes, care partners could also commit more of their energy and resources to providing a higher quality of life: “I’d far rather be putting my energy into making her life lovely” (CP 06).

Despite the anticipated benefits, participants expressed concern over the perceived finality of a decision to move the resident. Although LTC policies were adjusted in response to the pandemic to allow residents to move out of LTC temporarily, residents and care partners were skeptical that they would be permitted to move back in: “you were told you could take your loved one out of the home, and I don’t know if I trust that we will get them back in” (CP 10). Many residents had also previously spent months or years on the LTC waitlist and wished to avoid this reality in the future. Health professionals offered residents and care partners conflicting information on whether they could return to LTC post-transition.

The decision to move a resident out of LTC was expected to have indirect consequences on the lives of residents and care partners. For example, residents and care partners would need to adjust to new living arrangements. Care partners might also need to shift their work schedules or even quit their jobs to care for the resident. One care partner also believed a transition might also create new challenges for their relationship: “I think it’s going to put a strain on our relationship. I think them being with us 24/7 is too much” (CP 08). Several care partners were concerned about the impact that moving a resident would have on their life and wellbeing: “it would have been just too one dimensional for me, it would have been my entire life because he could not be left alone” (CP 03). For some, heightened caregiving responsibilities were a significant barrier: “we did a fairly extensive pro-con list as a family, and honestly, the biggest con was the fact that we’d be 24/7 caregivers” (CP 06). Care partners were also concerned about the impact that moving a resident would have on others in their life. Many of the care partners lived with spouses and were cognizant of their preferences: “Maybe if I was single I could have done it. But looking back, I think, like you had to be considerate of your partner, right” (CP 03).

Both response options were associated with positive and negative emotions. It was important for care partners to consider their mental health prior to moving a resident. Participants noted that either response option could lead to feelings of guilt and regret. For example, when considering leaving a resident in LTC one care partner stated: “I also knew if anything happened, I would have terrible, terrible guilt knowing that I didn’t do what I could have done” (CP 12). Conversely, participants might come to regret their decision if a transition went poorly. Moving a resident might also foster resentment; however, some care partners believed that the opportunity to care for the resident in their homes was a blessing.

## Discussion

This qualitative study explored how residents, care partners and health professionals made care transition decisions in LTC settings in Ontario during the COVID-19 pandemic. Data were analyzed through the lens of CDT [18], and presented according to its proposed three-stage process. In the first stage, participants assessed the severity of the crisis by drawing on a variety of comparative information and their emotional responses. Assessments were varied across participant groups, with care partners perceiving the highest severity and urgency to respond. In the second stage, participants considered potential response options and their feasibility. Two options were considered: leaving the resident in LTC or moving the resident into a home in the community. Although many residents and care partners wished that the resident could move to the community, it was often unfeasible. In the third stage, participants evaluated which of the response options was more desirable. They considered the benefits and drawbacks of each response for the resident and contemplated the broader impact of the care transition.

CDT was a useful framework to interpret participants’ decision-making processes. Theory brings value to qualitative research as it offers researchers different lenses that can be used to examine problems and social issues [40, 41] and focuses the researchers’ attention on specific variables of interest [42]. In this study, participants’ decision-making processes could be closely mapped to the three stages described in CDT. Each of the key constructs proposed in CDT also featured prominently in participants’ experiences suggesting a strong connection between the theory and this research. However, Sweeny (2008) posits that the function of CDT is twofold and involves both describing decision processes and predicting response choices to negative events [18]. For example, Sweeny suggests that “people might be more willing to invest in a solution when they feel a sense of responsibility for the negative event” [18; p.63]. In our study, care partners often expressed a deep feeling of personal responsibility for placing a resident in LTC and thus exposing them to heightened risks during the pandemic. Despite this, not all care partners who expressed this feeling opted to take action to change the care environment of the resident. Thus, while there may be many possible explanations for their varied responses the naturalistic focus of our research limited our ability to confirm or explore predicted responses.

A significant finding of this research was the critical role of emotion in crisis decision-making. In this study, participants experienced a range of negative emotions in response to the COVID-19 pandemic, including fear, anger, guilt, and regret. Participants also described a desire to achieve positive emotions through their decisions, including love, joy and gratitude. These positive and negative emotions impacted both their perceptions of the severity of the event (Stage 1) and how they evaluated their response options (Stage 3). Many participants commented on the influential role of emotion, stating the need to balance their emotional reactions and logical assessments in decision-making. Although CDT proposes that people may consider the potential emotional consequences of a response when making a decision [18], the broader impact of emotion on crisis decision-making is not explored. Other scholars have noted this limitation in traditional views of crisis decision making like CDT and suggested that “the complex interplay between cognitions and emotions can no longer be ignored in understanding information processing and decision-making during a crisis” [34; p.96]. Emotional reactions may play a particularly important role in crises [43], as uncertainty and time pressure may lead individuals to base decisions more on intuition [44] and emotions can help individuals save both time and energy [45]. Future researchers should account for and explore how emotion shapes decision-making in crisis events.

Although CDT offered a clear and straightforward lens to understand transition decision-making during the COVID-19 pandemic, critical attention should be paid to the often non-linear, dynamic and social nature of decision-making. To this end, extending CDT to incorporate properties of sensemaking may offer a more fulsome understanding of decision making in crisis events. Sensemaking theory was introduced by Weick in the 1970s to provide insights on how individuals and groups interact with their environments and with others to give meaning to, and make sense of, novel events [46, 47]. A core tenet of the theory is that sensemaking is “grounded in identity construction” and that people’s understandings and perceptions of the world are shaped by their personal identities and past experiences [46]. Thus, how people derive meaning in a crisis will differ and “may be motivated by goals other than increasing congruence with the data at hand” [48]. While CDT acknowledges the important role of past experiences when assessing the severity of an event, it does not explore the extent to which decisions may be driven by our actual or desired sense of self. For example, in the present study care partners often described the centrality of their identity as a caregiver in their desire to move the resident. In many cases, their decisions were motivated by both the dual goals of mitigating risk and acting in line with their personal identity and beliefs about caring for an aging loved one. Thus decision-making was intertwined with sensemaking, and responses were selected based on their meaningfulness to the individual and their personal views.

This research also sheds light on the added complexity of group decision-making in crises, an area that CDT does not explore in depth and where sensemaking may be of use. Mills’ (2010) discussion of sensemaking recognizes how meaning is situated within the broader social environment and shaped by power and privilege [46]. In contrast, Sweeny’s (2008) depiction of CDT [18] pays little attention to how social relations and power may shape decision processes. In this study there were clear power dynamics between members of different stakeholder groups with care partners often driving decision-making while health professionals advised. Notably, stakeholders from these groups often held different perspectives on the crisis with care partners perceiving the highest risk and health professionals the lowest. These differences in perspective may have influenced the stakeholders’ decision processes and preferred response options. Other research has shown group differences in how stakeholders perceived and responded to risk in LTC during the COVID-19 pandemic [49]. Recognizing these differences alongside associated power dynamics may be critically important to understand crisis decision-making in the future.

The type of care transition studied in this research (from LTC to community) has important implications for multiple stakeholder groups. For example, a decision to move the resident would impact the life of the care partner by increasing their caregiving responsibilities significantly and with limited resources or home care supports available. Additionally, health professionals’ accountabilities for the resident’s care would change as they moved between settings. Participants prioritized the perceived needs and preferences of the residents to the best of their abilities when making their decisions. Unfortunately, emerging literature on surrogate decision-making during the COVID-19 pandemic has shown that surrogates often have poor accuracy in predicting patient preferences [50]. These discrepancies appear relevant in this study, as some care partners described moving the residents despite their expressed preferences to stay in LTC. Further complexity is added when we consider that the consequences of these decisions operate beyond the resident to the care partners, family and broader health system. Thus, despite care partners’ and health professionals’ best intentions it would not be realistic for them to ignore the personal impacts of the response options during decision-making.

Shared decision-making may offer unique value in supporting the success of future care transitions in the LTC sector. Shared decision-making is a collaborative approach to care planning, whereby relevant stakeholders work together to consider the best available evidence and make a decision [51]. Although it is complicated to implement, it may be particularly beneficial in the context of care transitions because it can bridge multiple stakeholder perspectives [52]. However, research has shown that shared decision-making may be impeded during crisis events like the COVID-19 pandemic [53]. This may be due to the perceived urgency to make decisions quickly and limited opportunity to consult others [54]. In this study, participants often identified care partners as the main decision-maker regarding care transitions, with residents and health professionals involved more peripherally. In fact, from participants’ accounts it appeared that shared decision-making was often limited, and in many cases may not have occurred. While having a core decision-maker may be useful to expedite decisions in times of crisis, power imbalances between stakeholder groups can also impede shared decision-making [55] resulting in suboptimal decisions. Thus, it may be useful to consider tools to engage different stakeholder groups in rapid decision-making to improve transition outcomes.

### Limitations

There are a few limitations of this research to report. First, we chose not to ask participants to identify the specific LTC settings where their decision-making took place. Ontario’s LTC sector has been under considerable scrutiny throughout the pandemic, with several LTC locations being identified in the media for their poor standards of care and infection control practices. Therefore, to encourage participation and protect the identity of our participants and associate organizations, we chose not to collect this information in the research. However, in doing so, we forfeited the ability to reliably analyze differences in decision-making processes based on the extent or severity of crisis experienced by the settings. Second, few residents were successfully recruited to this research, limiting our understanding of their unique perspectives and decision processes. LTC residents often have cognitive impairment which complicates their inclusion in research. Additionally, many LTC settings in Ontario have gone through regular lockdowns where visitors are unable to enter the premises, and there was insufficient technology and infrastructure to enable virtual communication [56]. Recognizing the importance of residents’ perspectives to this research, we sought to obtain their perspectives through the views of others. Specifically, we asked care partners and health professionals to comment on what they knew of the residents’ perspectives, where possible. Third, due to the naturalistic nature of this research which explored decision-making in an ongoing crisis, we were unable to test the predictions proposed in CDT of crisis responses. Future research might consider recruiting a larger and more controlled sample population to test the accuracy of these predictions. Finally, deeper understanding of crisis decision-making may be achieved by collecting longitudinal interview data over time, rather than single interviews because decision processes and outcomes may differ depending on the status of the crisis event and availability of information.

## Conclusion

This research explored the decision-making of different stakeholders about care transitions in LTC settings across Ontario during the COVID-19 pandemic through the lens of CDT. Specifically, we sought to describe residents’, care partners’ and health professionals’ decision-making processes and how they were shaped by the pandemic. The results contribute novel findings related to the influential role of emotions on crisis decision-making and how different stakeholder groups perceived and responded to the crisis. Future research might focus on encouraging group engagement in crisis decision-making, identifying the variables most critical to predicting response choices, examining collective sensemaking, and exploring how decision processes evolve as crises unfold over time.

## Data Availability

All data generated or analysed during the current study are included in this published article.

## Abbreviations

LTC: Long-Term Care
CP: Care Partner
HP: Health Professional
CDT: Crisis Decision Theory
